# Monitoring SO_2_emission trends and residents’ perceived health risks from PGM smelting at Selous Metallurgical Complex in Zimbabwe

**DOI:** 10.1186/s12939-017-0696-6

**Published:** 2017-11-16

**Authors:** Patrick Gwimbi

**Affiliations:** Department of Environmental Health, National University of Lesotho, P. O. Roma, Maseru, 180 Lesotho

**Keywords:** SO_2_ emissions, PGM smelting, EIA, Communities located around smelters, Residents’ perceived health risks, Zimbabwe

## Abstract

**Background:**

Persistently high sulphur dioxide (SO_2_) emissions from platinum group metal (PGM) smelting pose a major threat to communities located around smelters. This paper examined SO_2_ emission trends, emission regulations and residents’ perceived health risks from exposures to such emissions at Selous Metallurgical Complex (SMC) PGM smelting facility in Zimbabwe.

**Methods:**

SO_2_ data from roof monitoring sites at the smelter furnace were aggregated into annual, quarterly and monthly emission trends from 2008 to 2015. The regulatory regime’s ability to protect human health from SO_2_ pollution in communities located around the smelter was examined. Questionnaire responses to perceived health risks from SO_2_exposure from 40 purposively sampled residents were assessed. The relationships between SO_2_ emission trends and residents’ self-reported health risks from exposure to SO_2_emissions were explored using STATA version 11. Descriptive statistics were used to illustrate SO_2_ emission trends and residents’ self-reported health risks from exposure to SO_2_.

**Results:**

Between 2008 and 2015, annual SO_2_emissions increased from 7951 to 2500 tonnes. Emissions exceeded the recommended standard limit of 50 mg/Nm^3^, presenting considerable adverse health risks to local residents. Concerns relating to inefficient environmental impact assessment (EIA) licensing system, poor monitoring and auditing by the environmental management agency, as well as non-deterring SO_2_emission exceedance penalties were identified as major drivers of emission increase. Thirty-two (80%) of the forty respondents perceived exposure to SO_2_ emissions as adverse and the cause of their illnesses, with coughing, nasal congestion and shortness of breath the most frequently self-reported symptoms.

**Conclusion:**

A set of legally-binding SO_2_emission standards supported by stringent EIA licensing arrangements for smelting industries are suggested for development and enforcement to reduce the SO_2_emission problem. Community participation in SO_2_emissions monitoring is also proposed as a core part of sustainable environmental management in communities located around smelters.

## Background

Recent analyses using epidemiological data has provided consistent evidence of damaging effects of sulphur dioxide (SO_2_) to human health [1–4]. Results of such studies have shown that exposure to SO_2_ account for increases in cardiopulmonary hospital admissions and mortality caused by reductions in lung function, asthma, emphysema and bronchitis [1, 3, 5]. There is also mounting evidence associating SO_2_exposure with ischemic heart disease, heart failure and stroke [5–7], and declining semen quality in males leading to infertility [4]. SO_2_toxicological data collected worldwide suggest that human fatalities can arise from short term exposure levels in excess of 1000 μg/m^3^ [8]. Human induced SO_2_emissions is the greatest threats to air quality and human health. Accordingly, there is merit in monitoring SO_2_emissions and investing in SO_2_emissions abatement technology in order to control its emission into the environment.

In recent years, platinum group metal (PGM) smelting facilities have been widely accused of generating extensive volumes of SO_2_ that expose communities located around smelters to health hazards [9, 10]. South Africa and Russia, the world’s leading PGM producers with over 88% of the global PGM output provide evidence of such polluting industries [11–13]. Tuovinen et al. [11] found SO_2_ emission in the Russian Kola Peninsula to be 600,000 tonnes per year, and concluded that this was one of the most polluted points in the world. Venter et al. [14] investigated SO_2_emissions in the Bushveld Igneous Complex in South Africa with significant PGM smelting activities and concluded that this was a SO_2_pollution hot spot. Increasingly, these heavily polluting smelting industries have become a significant problem in many developing countries, raising questions about the pollution haven effect on inhabitants of communities located around smelters who are more likely to suffer the detrimental health effects resulting from breathing polluted air.

From a theoretical point of view, some researchers [15–17] have proposed the pollution haven hypothesis to explain the migration of polluting industries from areas with stringent environmental regulations to those with lenient environmental regulations and policies. The pollution-haven hypothesis claims that stringent environmental regulations and high pollution abatement costs in developed countries drive multinational companies to establish plants in developing countries where regulations are lenient on polluters [15, 18]. An extensive literature has shown that in most African countries, economic growth concerns tend to override environmental pollution abatement considerations and environmental sustainability issues are rarely taken seriously [16, 19, 20]. Solarin et al. [16], for instance, in a study on Ghana, discovered a relationship between industrial carbon dioxide (CO_2_) emissions and environmental regulations, concluding that investing in pollution prevention technologies was outweighed by the short-term benefits accruing from increased production. The same conclusion was found by Riti and Shu [19] on Nigeria regarding emissions of CO_2_ by manufacturing industries, noting that less stringent regulations were a significant determinant attracting polluting industries in the country. In another paper, Sun et al. [20] proved that in a similar developing country like China less stringent environmental regulations were more attractive to polluting multinationals using CO_2_ emissions data. And, because of this perception, multinational companies have increasingly located to developing countries, citing lenient environmental regulations, cheap labour and natural resources and absence of costly pollution abatement technology as sources of comparative advantage [15, 16]. In Western Europe and North America, although there are few remaining SO_2_ emission threats, SO_2_is generally regarded as a problem that is carefully regulated, managed and watched over by those countries [21, 22]. Nevertheless, this decline is not homogenous across world, suggesting the possibility that environmental regulation affects the location of polluting industries. The concentration of polluting industries in countries with lax regulation imposes substantial health costs on such countries’ budgets.

The bulk of the population vulnerable to SO_2_emissions in developing countries live in the vicinity of PGM smelting facilities, and has limited information about its rights and capacity to defend itself or influence policy decisions [23]. Epidemiological evidence elsewhere shows adverse health effects of air pollution, including increases in morbidity and mortality from respiratory and cardiovascular causes [3, 5, 23]. Several of these studies have shown that there is a link between living near industrial complexes and occurrence of adverse health outcomes [24–26]. The Bench Marks Foundation [24] found out that exposed community members had a significantly high prevalence of cardiovascular and respiratory diseases as well as eye, nose, and throat irritations in Marikana, South Africa. Steyn [25] also reported increases in cardiovascular and respiratory diseases among residents in Rustenburg in South Africa where most PGM smelting facilities are located. Sulphur dioxide also causes acid rain that kills flora and fauna [26].

In Southern Africa, with the largest known PGM deposits in the world and increasingly fast growing smelting industrial facility population, communities living adjacent to such facilities are increasingly exposed to elevated concentration levels of SO_2_emissions, with numerous adverse human health effects [10, 14]. In this region, primary smelting of PGM ore concentrates is carried out by five multinational companies, namely Anglo Platinum, Impala Platinum, Lonmin Platinum, Northam Platinum, all of South Africa, and Zimabwe Platinum Mines (Zimplats) in Zimbabwe [10]. The multinational companies account for 100% of the PGM smelting that takes place in the region and are known for their adverse effects on human health and the natural environment [10].

The Great Dyke of Zimbabwe, with an estimated 4.4 billion tonnes of PGM ores, is the world’s second largest PGM resource after South Africa’s Bushveld Complex, and currently contributes 6% of the world’s PGM production [27]. Currently, three multinational firms operate along the Great Dyke, namely, Zimplats, Mimosa and Unki Platinum. The industry is one of Zimbabwe’s most important economic drivers, accounting for approximately 17% of the country’s total exports and 3.5% of the country’s GDP [27]. Selous Metallurgical Complex (SMC), located on the northern part of the Great Dyke is currently the only PGM smelter in the country (28). The smelter consist of a concentrate filtration plant, a drytech flash dryer, a modified elkem / hatch 13.5 MVA circular furnace, two 10 × 15 Peirce-Smith converters, and a matte granulation plant [13]. It uses a three-field electrostatic precipitator, supplied by ELB-Brandt, for removal of particulates in the gas streams from both the converter and furnace. Over the last few years, PGM smelting at the facility has increased significantly, emitting high volumes of SO_2_ into the environment. Concerns have been raised over the potential association between SO_2_ emission trends and adverse respiratory health effects including decrements in lung function. Evidence of the emission trends, abatement efforts and effects on the health of local communities is minimally known.

In this study, SO_2_ emission trends, regulations and technology used to control such emissions and residents’ perceived health risks from exposure to SO_2_ emissions at the Selous Metallurgical Complex PGM smelting facility in Zimbabwe are examined. Findings from this study may serve as an important base for further studies.

## Methods

A case study approach was adopted. SO_2_ emissions data measured and recorded from roof monitoring sites at the Selous Metallurgical Complex smelter furnace for the period 2008 to 2015, regulations used to control the emissions and local residents’ self-reported symptoms of SO_2_exposure were examined. The data was both quantitative and qualitative.

### Study site

The study was confined to a 10 km radius area from Selous Metallurgical Complex (SMC) PGM smelting facility. SMC is located on the northern part of the Great Dyke, 80 km south west of Harare, Zimbabwe’s capital city [28]. It is the only PGM smelting facility in Zimbabwe.

SMC experiences a convective-rainfall type rainy season from the beginning of December to mid-March followed by a period of prolonged dryness. The mean annual rainfall is 876 mm. The vegetation is generally grassland with open shrub-land and woodland forest on adjacent riverine areas [29].

The study population consisted of respondents engaged in diverse activities including farmers, formal and informal traders and farm workers and other people who frequently visited the area in search of employment. Farming activities are generally associated with cash crops and vegetables. Formal and semi-formal settlements were observed within a kilometer from the smelting facility. A shopping centre with a motel, administrative police station, fuel service station and frequented by informal traders along the Harare-Bulawayo highway was observed. Forty residents were purposively sampled taking into consideration the whole geographical area, local leadership, different activities and gender.

### SO_2_ emission trends and regulation control measures

SO_2_ emissions data measured and recorded from roof monitoring sites at the smelter furnace were aggregated into annual, quarterly and monthly emission trends from 2008 to 2015. Environmental regulations controlling emissions were reviewed. The smelting company’s environmental management plans, environmental monitoring reports and annual sustainable development reports were also evaluated.

### Survey of residents’ perceived health risks from exposure to SO_2_

A cross sectional study was conducted in communities within a 10 km radius from Selous Metallurgical Complex smelting facility. Prior to the questionnaire survey, a human subjects research protocol was sought for and approved by Zimbabwe’s Environmental Management Agency (EMA) and Ministry of mines and mining development in Zimbabwe. Each survey lasted approximately 30 min.

Respondents were given an information sheet explaining the purpose of the study and the method of data collection that was being used. All the respondents were given a consent form to sign if they agreed to participate in the study. They were also informed that they were free to withdraw from the study or not answer questions they did not feel like addressing. The researcher safeguarded respondents’ identities and responses from public disclosure by ensuring anonymity on the administered questionnaires. In addition the consent forms, the researcher also clarified how the collected information would be used.

The questionnaire collected information on demographic characteristics of the respondent, self-reported health symptoms due the smelting facility activities and perceived causes. The questionnaire was translated into the local language with back translation into English in order to ensure that the local language version.

### Data analysis

Research findings were explorative and explanatory and demanded the use of both quantitative and qualitative data analysis techniques. Time-series graphs were used to explore SO_2_ emission trends, with the unit of observation being tonnes for the period 2008 to 2015. The analysis assessed the annual, quarterly and monthly emission variations. Information from residents was analyzed using the statistical software STATA version 11. Descriptive statistics were used to illustrate the SO_2_emission levels and perceived health impacts on local residents. Graphs, means and tables were used to describe variables.

## Results

### Trends in SO_2_ emissions (2008–2015)

**Fig. 1:**
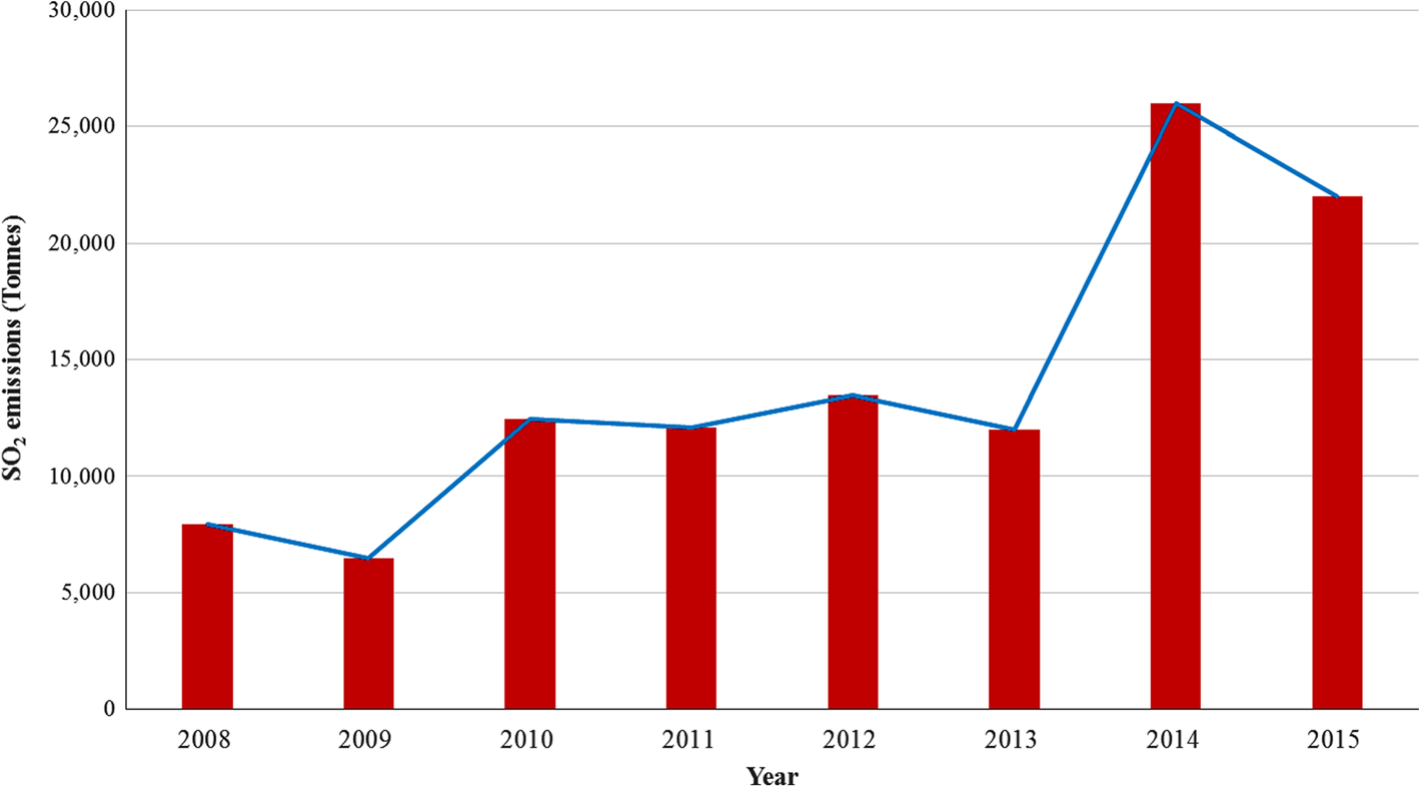
Annual SO_2_emissions at Selous Metallurgical Complex (2008–2015)

Annual SO_2_ emissions at the SMC PGM smelting facility from 2008 to 2015 are shown in Fig. 1. Emissions increased from 7951 tonnes to 25,000 tonnes over the study period. Between 2008 and 2009 there was an 18.8% reduction in annual emissions due to furnace breakdown incidences. Emissions increased by 92% from 2009 to 2010. From 2013 to 2014, SO_2_emissions increased by 116%. Over the same period PGM ore concentrate deliveries for smelting increased by 27% [30].

**Fig. 2:**
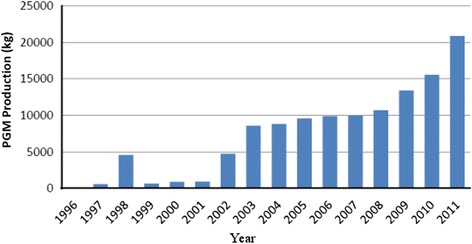
Trends in PGM production in Zimbabwe (2001–2013)

The inter annual SO_2_ emission increases were consistent with increasing volumes of PGM ores delivered for smelting (Fig. 2) and high sulphide mineralogy of the PGM concentrate [30, 31]. The smelting facility’s annual report [30] also noted that the mining expansion operation programme resulted in increased PGM ore output at Ngezi mine leading to more ore deliveries to SMC. Episodic declines in emissions were attributed to furnace breakdown incidences [30, 31].

**Fig. 3:**
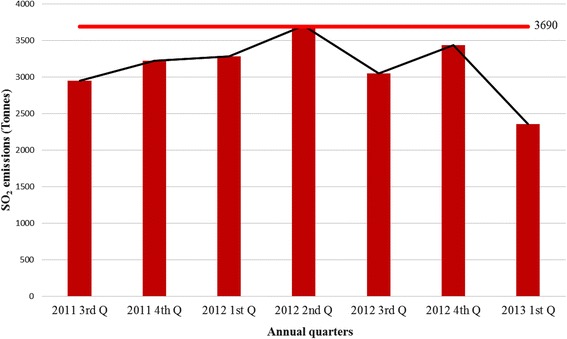
Quarterly SO_2_emissions at Selous Metallurgical Complex (2011–2013)

Concerning, the inter-quarterly SO_2_emissions, an increasing trend (Fig. 3), with episodic declines due to furnace breakdown incidences was also observed. The emission increases were however within the smelting company’s permissible emission limits issued by the country’s environmental management agency (EMA) according to Statutory Instrument (SI) 72 of 2009 which provided for the establishment of emission standards for various pollutants [32] as shown in Fig. 3.

**Fig. 4:**
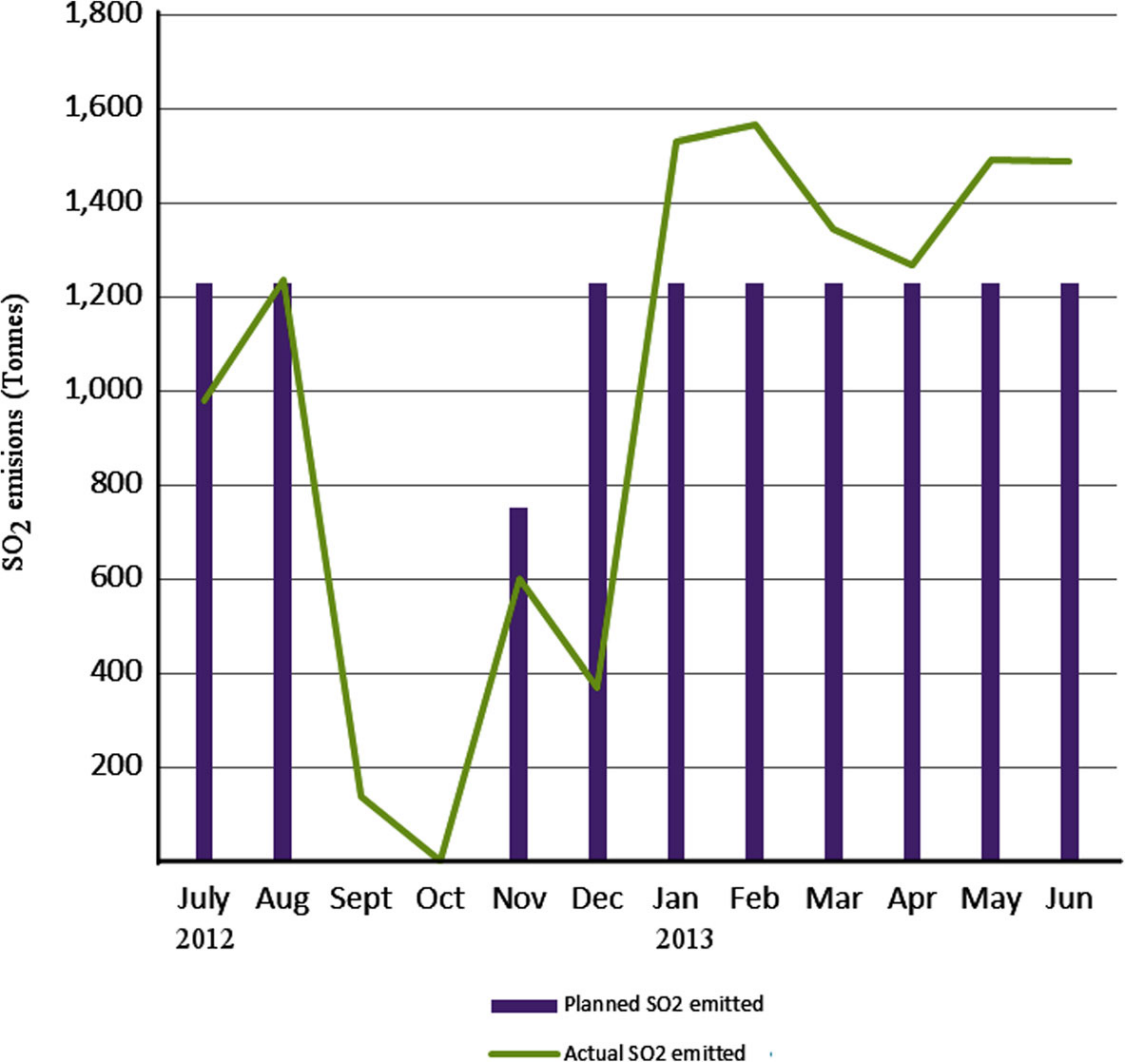
Monthly SO_2_ emissions at Selous Metallurgical Complex (2012–2013)

Inter-monthly SO_2_emissions between 2012 and 2013 were characterised by episodes of both increases and declines (Fig. 4). From Fig. 4 it can be observed that SO_2_emissions for the reference months and year were considerably high and greatly exceeded the limit values that were expected to be met in 2013 in most cases according to SI 72 of 2009. Emission reductions due to furnace breakdown incidences were recorded between August and December 2012.

Overall, the results indicated emission exceedances of the SI 72 of 2009 legislated limit of 50 mg/Nm^3^ at SMC, due to high sulphide mineralogy content of the increasing volumes of PGM ores delivered for smelting. The trend in emissions is expected to continue in the foreseeable future under the prevailing conditions. This indicates that maximum smelting facility operation emissions produced SO_2_concentrations that were above regulatory standards designed to protect public health.

### Current regulatory practices controlling SO_2_ emissions

SO_2_ emissions from SMC smelting facility are regulated by the country’s Environment Management Act (Chapter 20:27) of 2002, sections 55–86. Section 9 of the Environmental Management Act (Chapter 20:27) gives the Environmental Management Agency (EMA), the parastatal created to regulate, monitor and manage environmental issues in Zimbabwe, the mandate to assist in the management and protection of environmental issues in Zimbabwe [32]. Statutory Instrument (SI) 72 of 2009 of the Act provides for the establishment of emission standards for various pollutants. The instrument addresses the reduction of risks from exposure to single pollutants including SO_2_ on the basis of ambient standard limits set. For SO_2_ the emission limit is 50 mg/Nm^3^. The objective of SI 72 of 2009 is to provide for prevention, control and abatement of air pollution to ensure clean and healthy ambient air by providing for the establishment of emission standards [32, 33].

Emission limits are categorised by colour coding into: blue, for which pollution is environmentally safe; green, a low environmental hazard, yellow, medium environmental hazard; and red, high environmental hazard, based on concentration of the emission and mass flow [32]. The pollution fines for categories green, yellow and red bands range from USD$340, $490 and $640 respectively [32]. Any emission above the red class upper threshold value is not licensed.

The current pollution licensing system allows SO_2_emissions at levels likely to be dangerous to human health in communities located around the SMC smelter. Results from quarterly monitoring reports showed that emissions were generally in the red license category according to SI 72 of 2009. Over the years, the SO_2_ emission standard of 50 mg/Nm^3^ has been exceeded [30, 31, 34]. These results suggest negative health impacts of the high levels of SO_2_pollution in the area. In light of this observation, the smelter is operating in contravention of the pollution limits set in its operating licence. It appears that the current emission limit at which the smelting facility operates is not adequate to prevent pollution at levels harmful to human health.

Zimbabwe’s Auditor-General Report of 2015 indicate that EMA’s pollution penalty system is non-deterrent [32], casting doubt on whether the fines imposed by EMA on the smelter are sufficient to deter it from emission exceedances. The audit results suggest that repeated pollution exceedences and payment of the same fine by several offending mines continued [32]. Thus, environmental offenders were finding it rather cheaper to pollute and degrade the environment because it seemed easier for them to pollute and pay. The report also noted that when offenders faced prosecution the courts were ineffective as environmental offences were not given priority in comparison to other criminal cases [32].

In addition to SI 72 of 2009, another instrument, SI 7 of 2007 which provides for the procedures for conducting EIA and issuance of EIA licence was found relevant to this study. This instrument exerts its effect to the smelting facility by specifying the measures for minimising and where possible eliminating adverse effects and enhancing positive ones. The smelting company submits its environmental management plans and quarterly emission monitoring reports to EMA which is empowered by the Environment Management Act (Chapter 20:27) to penalise polluters. Section 26(1) of SI 7 of 2007 provides levels of fines chargeable ranging from level 1 to level 14. Level 1 is the minimum charge which is US$5.00 and level 14 is the maximum currently fixed at US$5000.00 [32]. EMA is also expected to carry out bi-annual environmental audits at the facility to ensure compliance with conditions set out in the EIA certificate.

The 2015 auditor-general’s report found the capacity of EMA to audit and monitor emissions inadequate [32]. EIA follow-ups on whether environmental management plans provided in the environmental impact statements (EISs) were implemented by project proponents were not fulfilled [32]. The report revealed shortcomings in the monitoring of environmental issues, including failure to control miners operating without EIA certification, inadequate monitoring of EIA certificate renewal, failure to monitor emissions and implement adequate measures against environmental offenders, poor maintenance of records and inadequate human resources [32]. EMA acknowledges this in its response to the findings, attributing it to the unavailability of enforcement staff due to government freeze on recruitment by government institutions [32].

EIAs for mining and related projects have had their effectiveness questioned for lacking scientific rigour on post project approval environmental management and monitoring matters [32]. The most frequently mentioned problems were poor quality of monitoring programmes and translation of recommended mitigation measures into decision-making.

Zimbabwe has been an active participant in both United Nations Framework Convention on Climate Change (UNFCCC) and Kyoto Protocol. In spite of this, the country is yet to transform these international legal obligations into its domestic laws and practice [35].

The conclusions drawn regarding the effectiveness of legislative measures in controlling SO_2_ emissions is that the regulations were not effective. SO_2_ emission limit set according to SI 72 of 2009 was exceeded and fines repeatedly paid as they were not deterrent.

### SO_2_ emission abatement at the SMC smelting facility

The technical options used to control SO_2_ emissions at the SMC smelting facility were reviewed. A furnace off-gas sampling study undertaken by Mabiza et al. [2] indicate that there is no sulphur abatement technology used to control SO_2_ emissions at the smelter. Resultantly, emissions are unsustainably high, a fact acknowledged in the smelting facility’s annual reports.

There is nonetheless suggestion of sulphur abatement technology being installed at the smelting facility in future. An acid plant route consisting of a tail gas, an acid plant and converter has been suggested as the option for lowering SO_2_ emissions due to its perceived benefits of handling high SO_2_ concentrations of converter off-gas [36].

### Residents’ perceived health risks from exposure to SO_2_ emissions

#### Description of the sample

Forty respondents participated in the study. The participation rate was 100%. The mean age was 39 years. Majority of the respondents were males (60%), as a result of the fact that these were looking for employment opportunities at the smelting facility (Table 1).

**Table 1:** Socio-demographic characteristics of the respondents

Variable studied		Percentage of the sample (*n* = 40)
Sex	Male	60
	Female	40
Age group (years)	≤30	37.5
	31–40	22.5
	41–50	20
	51 and above	20
Duration of stay (years)	<1	2.5
	1–5	20
	6–10	10
	11–15	22.5
	>15	45
Marital status	Single	10
	Married	75
	Divorced	7.5
	Widowed	7.5
Highest level of education attained	No schooling	5
	Primary	45
	Secondary	50

From Table 1, 39 of the 40 respondents (97.5%) had stayed in SMC area for at least five years and thus had more experience with the surrounding environmental conditions. This supports their knowledge of prevalence of risks from exposure to SO_2_ emissions from the smelting facility.

From Table 1, the married respondent population was the highest with 30 respondents being married (75%) while unmarried and widowed respondents were 4 (10%) and 3 (7.5%) respectively. The majority of the respondents had secondary education (50%) while 18 (45%) had primary education.

The number of the respondents who depended on farming alone was 20 representing 50% of the total respondents interviewed while those with informal business related to farming were 13 representing 32.5% of the total number of respondents interviewed, followed by the rest engaged in non-agricultural activities.

#### Residents’ self-reported health risks from SO_2_exposure

**Fig. 5:**
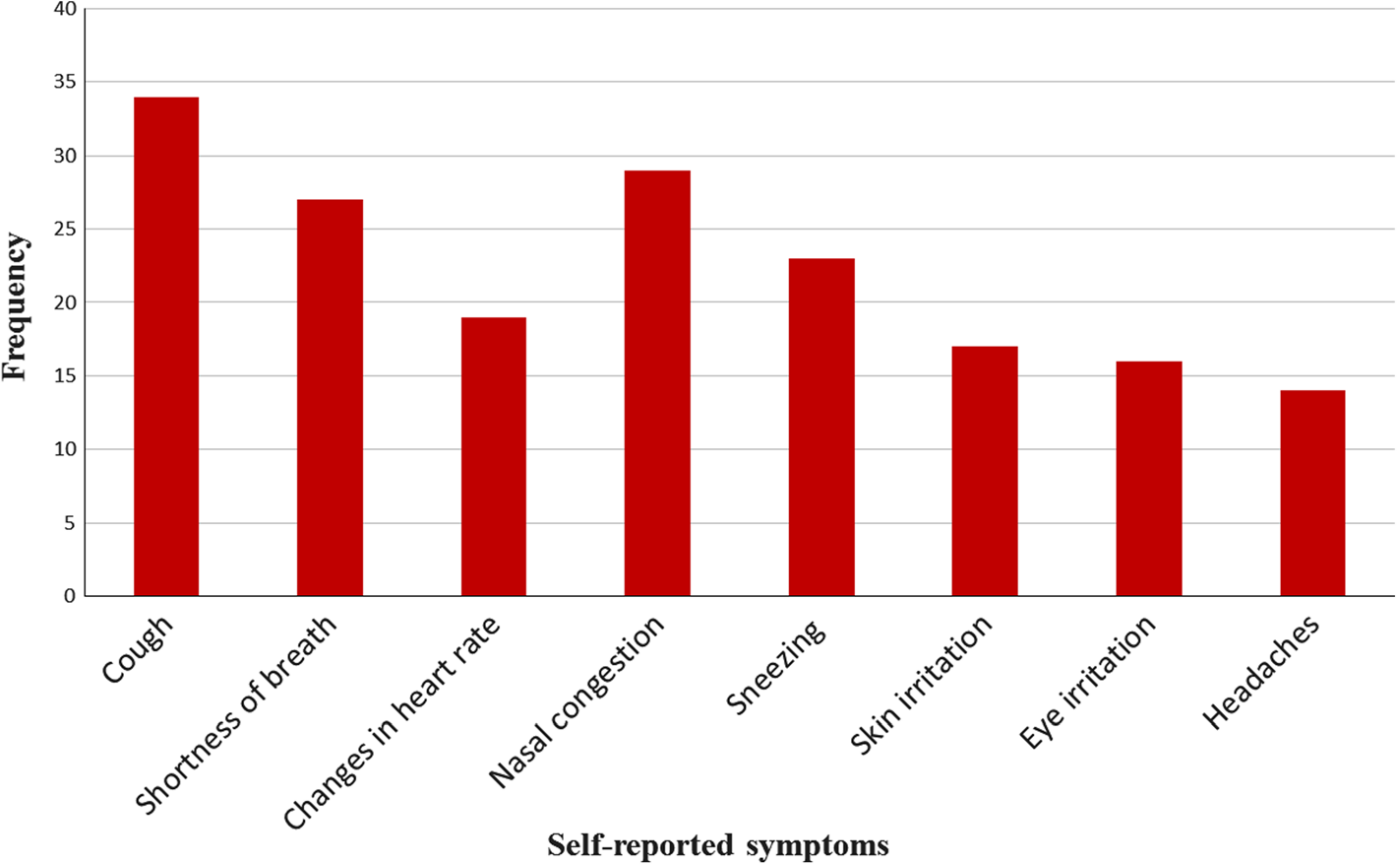
Self-reported symptoms due to exposure to SO_2_emissions

Respondents’ self-reported symptoms due to exposure to SO_2_emissions are shown in Fig. 5. Coughing, nasal congestion and shortness of breath were high the most frequently reported symptoms.

Proximity to the SMC smelting facility had a strong influence on the frequency of experiencing symptoms shown in Fig. 5. Out of the 20 respondents residing within a radius of 5 km from SMC (86.7%) reported experiencing five of the most frequently reported symptoms shown in Fig. 5 (coughing, nasal congestion, and shortness of breath, sneezing and changes in heart rate).

Coughing, nasal congestion, and sneezing were the three most frequently mentioned symptoms mentioned by respondents at least every six months (31%, 24% and 18% respectively) (Table 2).

**Table 2:** Self-reported frequency of illness among residents living within the SMC area

Reported symptom	Frequency					
	Every day (%)	Once a week (%)	Once a month (%)	Once every 3 months (%)	Once every 6 months (%)	Not sure (%)
Cough	0	3	21	6	1	9
Shortness of breath	0	1	13	2	1	10
Changes in heart rate	0	0	4	7	2	6
Nasal congestion	1	11	9	3	0	5
Sneezing	5	8	5	0	0	5
Skin irritation	0	1	5	6	0	5
Eye irritation	0	1	3	5	1	6
Head ache	0	0	6	2	0	6

#### Residents’ perceived sources of health risks

**Fig. 6:**
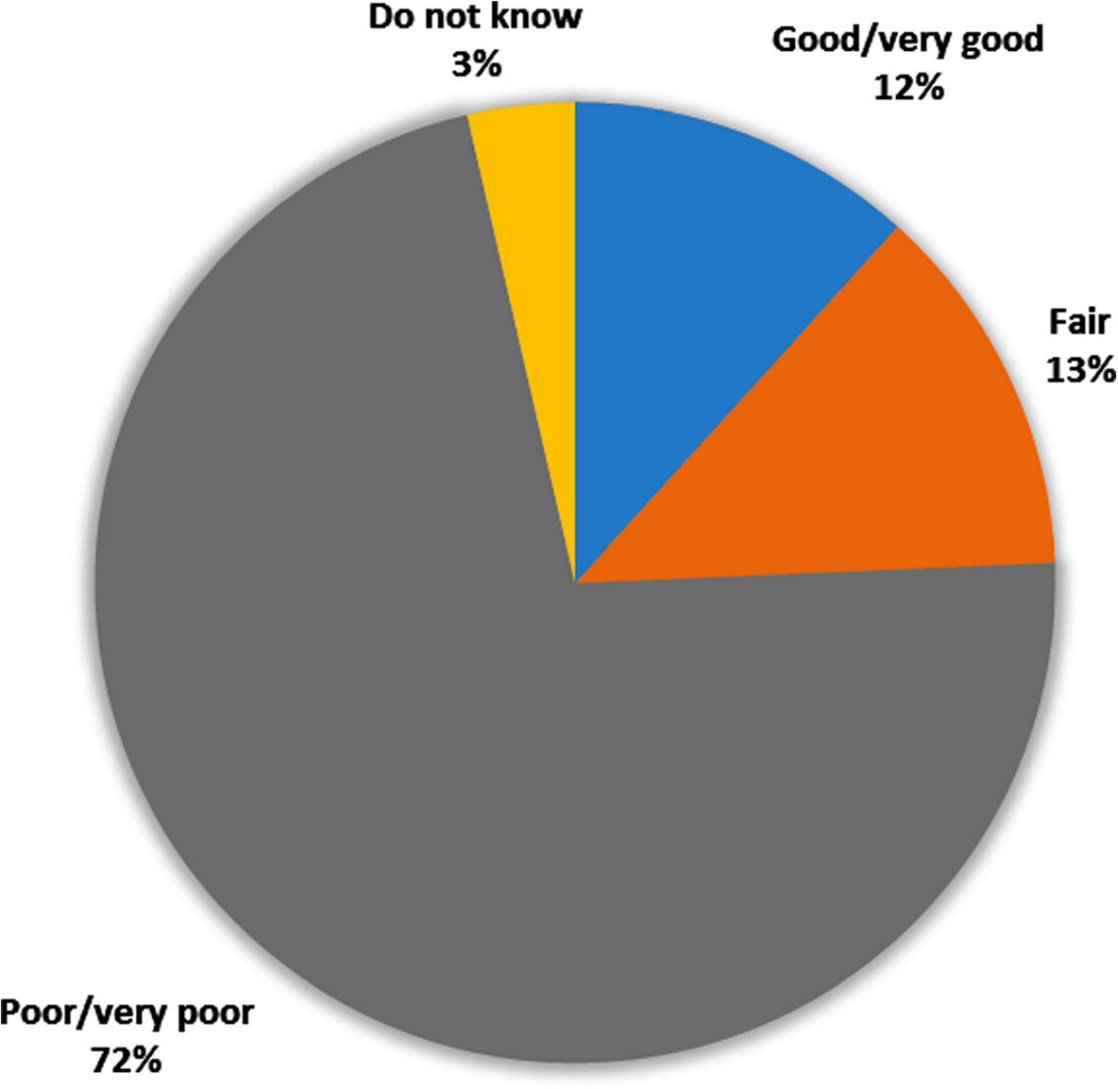
Self-reported perceptions about air quality surrounding SMC

Thirty two out of 40 respondents (80%) identified SO_2_emissions from the SMC smelting facility as the source of their health problems (Fig. 6). Twenty nine respondents (72%) classified the air quality in areas surrounding SMC as at least poor (Fig. 6). The duration of one’s stay, age, gender and marital status did not influence the respondents’ perceived sources of health risks in the area.

## Discussion

The findings of this study indicate that SO_2_ emission trends at the SMC smelting facility are increasing, posing health risks to communities located around the facility as well as underpinning a range of issues centred on smelting in Zimbabwe. The results also indicate the effectiveness of legislative measures being used to control SO_2_ emissions. Given the projected increasing demand for PGMs the negative environmental health effects of SO_2_ emission are likely to increase in future.

The above results suggest that there is a possibility that Zimbabwe is a pollution haven. One implication of the pollution haven hypothesis is public health concern from exposure to SO_2_ emissions [37]. The current SI 72 of 2009 regulatory instrument allows the smelting facility to emit SO_2_ in the red air emission category considered presenting high risks to the environment and human health. Though, at this stage, without knowing specific exposure ranges for the public and the associated rates of diseases that occur within those ranges need further research, the health of exposed communities located around smelters is at risk.

Stimulating economic growth and development could be playing a role in protecting the smelting facility. The acknowledgement by Zimbabwe Auditor-General Report that EMA’s pollution penalty system is non-deterrent despite emission limit exceedances suggest that the smelting facility might be finding it profitable to continue exceeding SO_2_ emission limits while paying the small fine. Prior studies on pollution haven hypothesis have also reported that multinational firms, particularly heavily polluting ones are in investing in environments with such less stringent regulations [16, 38, 39]. Petterson and Soderholm [38] for example, suggest that in the European Union, subject to issuance of smelting operating license, the use of best available pollution abatement techniques in accordance with the industrial emission directive is a condition that must be met by the industry intending to invest. Several studies exploring the deterrent effects of environmental sanctions have found that criminal sanctions significantly deter both penalized as well as non-penalized facilities [39]. According to Lynch et al. [39], when penalties exceed rewards, the offense should be deterred. Empirical results suggest that non-fined companies reduce their violation rates by two thirds following the fining of an offending company [39]. These findings seem to support Petterson and Soderholm [38] studies that observed deterrence effects associated with stringent regulatory sanctions.

EIA is established as a tool that promotes sustainable development [40]. In Zimbabwe EIA is mandatory. The current EIA in Zimbabwe heavily focuses on compliance with procedural requirements. Nonetheless, adverse health impacts of SO_2_ emission on people living in communities located around the smelting facility were reported, questioning the substantive effectiveness of EIA regarding community exposures to SO_2_ emissions. Substantive effectiveness examines if EIA actually reduces negative environmental impacts [40]. This idea includes sustainable development [40]. Loomisa and Dziedzic [40] acknowledge that less common in literature has been studies evaluating the substantive effectiveness of EIA. In this study, this is corroborated by the Zimbabwe auditor general’s report of 2015 that acknowledges that EIA follow-up on SO_2_emission exceedances and their impact on public health were missing. Previous studies have argued for community participation in emissions monitoring to ensure sustainably healthy communities [41]. As communities face increasingly SO_2_ related problems, policies that improve public health and enhance the quality of life for community residents are needed. The Bench Marks Foundation [23] suggests that community participation in SO_2_ emissions monitoring is critical in promoting sustainable communities surrounding polluting industries.

In light of the above, the current regulations are not adequate to prevent SO_2_ emissions at levels harmful to human health. A significant problem with the current emission controls relates to the fact that they are too lenient, and fines not sustainable in addition to not being sufficiently enforced. The current approach also tends to ignore the impacts of SO_2_ emissions on communities in close proximity to polluting sites. This approach fails to address the community sustainability issue when monitoring and assessing the substantive effectiveness of EIA in controlling SO_2_ emissions. It is well established in literature that substantive effectiveness is EIA’s long term goal [41]. SO_2_ emissions abatement technology such as a sulphuric acid plant has been suggested as the most effective means of capturing the sulphur from smelters [42]. Monitoring the results these exposure reduction strategies is critical, so that success of the programme can be assessed. This allows particular focus on cases where targets are not being met. This study proposes legally-binding SO_2_ emissions abatement technology that is supported by stringent environmental regulations. This was found lacking in this study.

## Conclusion

This paper investigated SO_2_ emission trends and its perceived health risks on communities located around Selous Metallurgical Complex PGM smelting facility in Zimbabwe. SO_2_ data from the roof monitoring sites of the smelter furnace, environmental regulations and technologies used to control SO_2_ emissions and residents’ self-reported health risks from SO_2_exposure were examined. The recommended SO_2_ emissions of 50 mg/Nm^3^ were exceeded. Emission increases were largely attributed to inefficient environmental impact assessment (EIA) licensing system, poor monitoring and auditing by EMA, as well as non-deterring SO_2_ emission exceedance penalties imposed on offenders. Communities located around smelters are adversely impacted by SO_2_ emissions, with respiratory diseases the dominant symptom. Legally-binding emission standards developed and enforced through stringent licence arrangements are proposed.

### Limitations of the study

This study acknowledges some shortcomings of its data. The study used SO_2_ emission based on data availability, not represent the same level throughout the geographical area studied. The small respondent sample in the survey may call for a bigger sample in future to be conclusive of the survey results.

## Abbreviations

EIS: Environmental Impact Statement
EMA: Environmental Management Agency
GDP: Gross Domestic Product
PGM: Platinum group metal
SMC: Selous Metallurgical Complex
SO_2_: Sulphur dioxide
UNFCCC: United Nations Framework Convention on Climate Change
Zimplats: Zimbabwe Platinum Mines
